# Discovery and biogeochemistry of asphalt seeps in the North São Paulo Plateau, Brazilian Margin

**DOI:** 10.1038/s41598-018-30928-2

**Published:** 2018-08-22

**Authors:** Kai Jiang, Jing Zhang, Akihiro Sakatoku, Shota Kambayashi, Toshiro Yamanaka, Toshiyuki Kanehara, Katsunori Fujikura, Vivian Helena Pellizari

**Affiliations:** 1 Graduate School of Science and Engineering, University of Toyama, Toyama, 9308555 Japan; 2 Graduate School of Natural Science and Technology, Okayama University, Okayama, 7008530 Japan; 3 School of Marine Resources and Environment, Tokyo University of Marine Science and Technology, Tokyo, 1088477 Japan; 4 Department of Marine Biodiversity Research, Japan Agency for Marine-Earth Science and Technology, Yokosuka, 2370061 Japan; 5 Instituto Oceanográfico, Departamento de Oceanografia Biológica, Universidade de São Paulo, São Paulo, 05508120 Brazil

## Abstract

An initial multiple biogeochemical dataset was acquired from the first discovered asphalt seeps in the Brazil margin during deep-sea dive surveys in 2013 using a manned submersible. These surveys were conducted on the outer escarpment of the North São Paulo Plateau. Sediment cores taken from the submersible were processed for pore water and sediment biogeochemistry. The silica concentration, as a chemical geothermometer, showed a steep gradient in the pore water, which indicates the possibility of an active brine system operating in the seepage area. Rare earth elements were used as powerful tracers of chemical processes. Low rare earth element concentrations in both asphalt and Fe-Mn oxyhydroxide-phase sediments suggests that rare earth elements were released during the oil fractionation and biodegradation processes and further depleted under the reducing environment. The main bacterial communities of the sediment were Proteobacteria in the asphalt sites, while at non-asphalt sites, the main bacterial communities of sediment were Firmicutes. Stable carbon and nitrogen isotopes were used to determine the food sources of the heterotrophs, and results suggest that asphalt probably provides a carbon source for these benthic animals. This study may provide useful information to clarify the impact of heavy hydrocarbon seepage on the marine ecosystem.

## Introduction

The significance of research on petroleum seeps has been rapidly and widely recognized, and large numbers of petroleum seeps have been discovered in the world’s oceans^1^. Petroleum seeps can serve as direct indicators of gas or oil accumulations^2^. Furthermore, it has been proven that petroleum seeps can support chemosynthetic communities^3^, which opens a new window for tracing the early evolution of life on Earth^4^. In addition, petroleum and other chemicals released into the ocean not only influence the marine environment and materials circulation but also affect the global climate^5,6^. Petroleum seeps include light hydrocarbon (gases such as methane) and heavy hydrocarbon seeps (oil seeps). Heavy hydrocarbon seeps are less common than light hydrocarbon seeps, although they account for 47% of all oil input to the ocean^7^. Our understanding of heavy hydrocarbon seeps is still limited and superficial. The most prominent seep sites are located in the Gulf of Mexico^8^, off the southern California coast^9^, the eastern Black Sea^10^, and the Angolan margin^11^. The heavy hydrocarbon seeps in the Gulf of Mexico and off of southern California have been the most reported and investigated. Approximately 95% of oil annually released into the Gulf of Mexico waters is from natural oil seeps^12^. The seeps in the Santa Barbara Channel are known as spectacular marine hydrocarbon seeps^9^. The heavy hydrocarbon seeps in the Angolan margin were discovered in 2008, leading to the first description of heavy hydrocarbon seeps in the South Atlantic and on the eastern Atlantic continental margin^11^.

**Figure 1:**
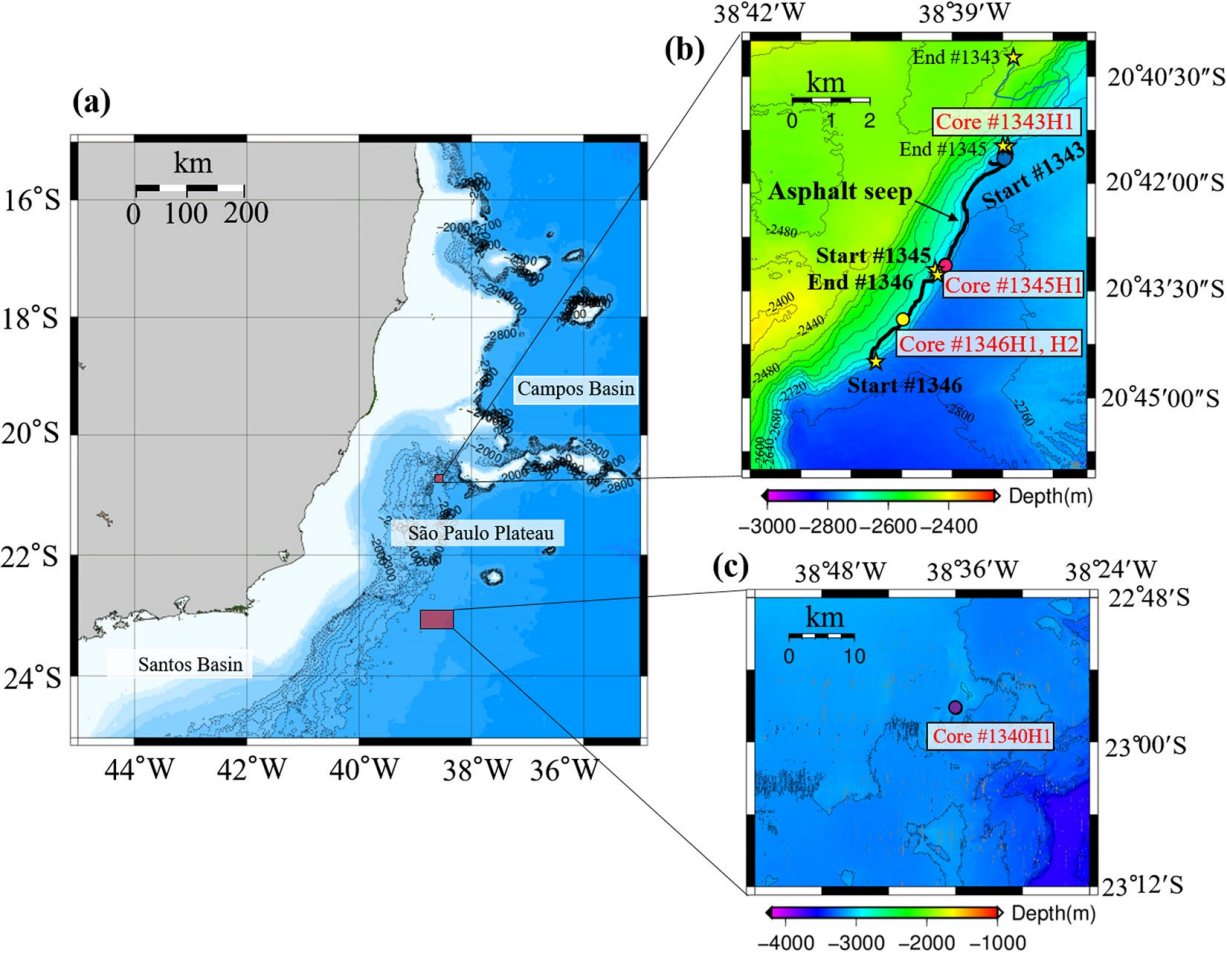
Area of the diving survey at the São Paulo Plateau off the Brazilian margin. Yellow stars show the start and end points of each dive, and red points show the sites of sediment core samples. Base maps were generated using Generic Mapping Tools (GMT 4.5.6), https://www.soest.hawaii.edu/gmt/.

On the opposite coast of the South Atlantic, the Brazilian margin has large oil and gas reserves and abundant diapiric salt structures^13^. This margin is a place where petroleum seeps may occur. However, no seep related to petroleum was reported there until 2013. The Japan Agency for Marine Earth Science and Technology launched the QUELLE2013 project in 2013, which was an around-the-world voyage by the SHINKAI 6500 (a manned submersible) aimed at studying the extreme habitats of the oceans near the limits of life, thus revealing the survival strategies of life on Earth under extreme conditions. The Iatá-piúna cruise was part of this project, and a deep-sea survey was first conducted in these waters using the manned research submersible SHINKAI 6500. Selection of the diving sites was performed on the basis of a 2D seismic profile. Before the diving surveys, we conducted multichannel seismic surveys and observed a 200-m escarpment on the seafloor relief, indicating the existence of geologic faults and salt diapirism. The dive surveys were performed along the escarpment. In total, nine dives were made in the São Paulo Plateau during the survey (Fig. 1a). During three of these dives in the North São Paulo Plateau, identified sequentially as #1343, #1345 and #1346, a huge black mass of lava-like material appeared on the seafloor. We eventually determined that the black material was asphalt according to later hydrocarbon composition analysis^14^. This was the first discovery of natural asphalt seeps in the Brazilian margin, which is one of the relatively less well-studied types of heavy hydrocarbon seepages. The asphalt seep was first discovered during dive #1343, then southward to #1345, and ending at #1346, for a total length of approximately 5.6 km (Fig. 1b).

**Figure 2:**
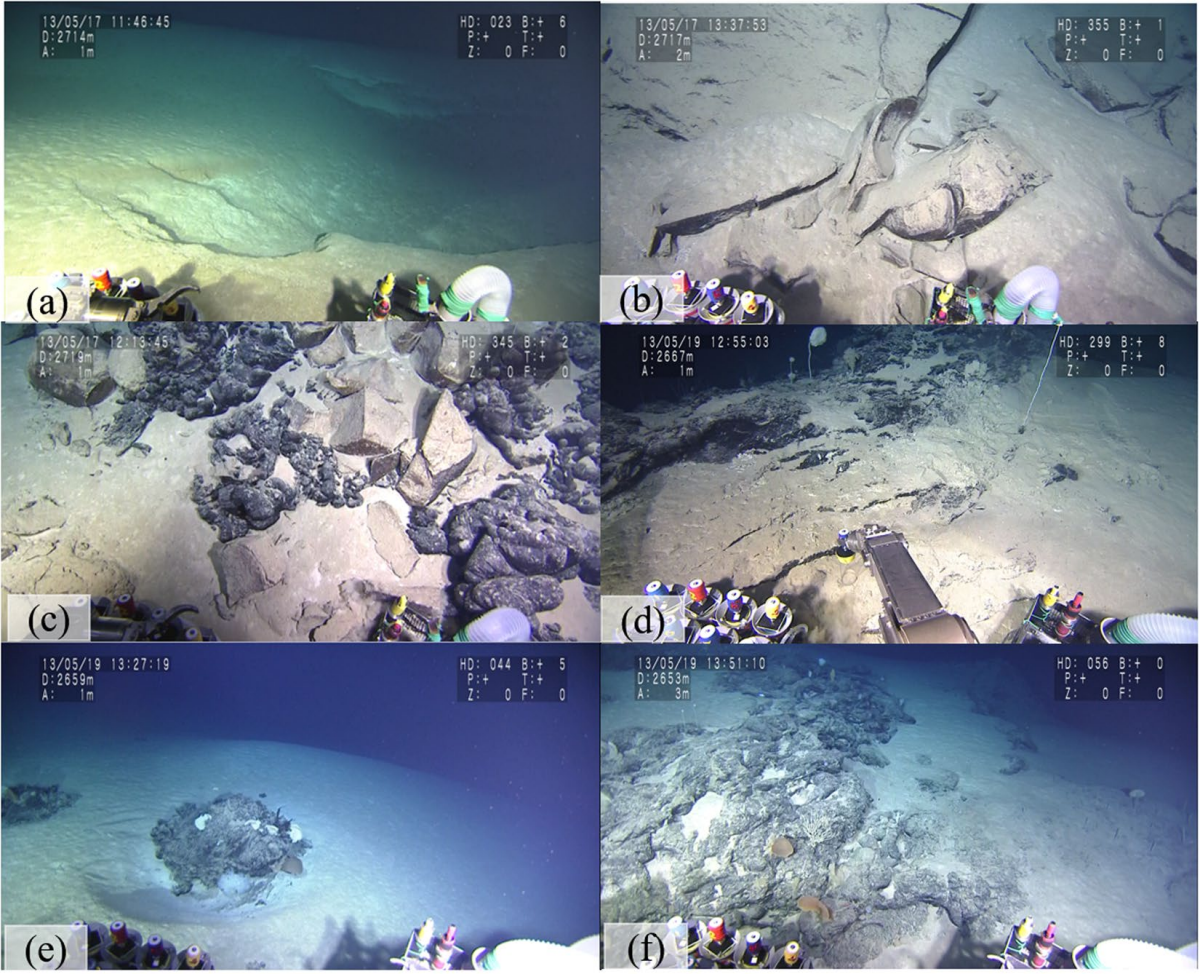
Photos taken from asphalt seep sites during the deep-sea dives in the São Paulo Plateau, Brazilian margin. (**a**) Seafloor covered by brown mud (dive #1345). (**b**) Mudstone rocks on the seafloor (dive #1345). (**c**) Seeped asphalt over rock outcrops (dive #1345). (**d**) Core sampling by the manipulators of the HOV (dive #1346). (**e**,**f**) Sponges associated with asphalt (dive #1346). Photos were taken using the high-resolution color TV cameras (NEC NC-H1000, Tokyo, Japan and Sony FCB-H11, Tokyo, Japan) installed on the submersible SHINKAI 6500 of the Japan Agency for Marine-Earth Science and Technology (JAMSTEC).

The deep-sea survey in this study confirmed previous study results indicating that the surface of the São Paulo Plateau is irregular largely because of abundant diapiric structures^15^. The topography along the dive track is complicated because it includes large rock outcrops, deep valleys, steep cliffs, sandy plains and gentle slopes. The area’s seafloor is typically covered by brown mud (Fig. 2a) with bioclastic fragments. Mudstone (Fig. 2b) composes the outcrops and is covered by black manganese oxide, and no pockmarks were observed during the observation^14^.

Asphalt (Fig. 2c) covers the seafloor in different types of extrusion forms, including reticulate filling types in the fissures of mudstone outcrops, linearly extruding types, lump types, scattered-stick types and mound types. More detailed descriptions are given by Fujikura *et al*.^14^. The asphalt was shown to be heavily biodegraded. Well-preserved terpanes and steranes are present in the samples collected during dive #1345, while samples from dives #1343 and #1346 show elevated proportions of biodegradation-resistant diahopanes^16^. This indicates that asphalt from dive #1345 had a relatively lower degree of biodegradation^16^. The absence of the 25-norhipane series in the asphalt sample from dive #1345 suggests that biodegradation occurs at the seafloor rather than in a reservoir^16^. Moreover, the asphalt shows a high C35 homohopane index, indicating that it is a carbonate source rock^16^.

To gain further understanding of these asphalt seeps, we took sediment cores from the asphalt seep sites and obtained the first biogeochemical data on the region’s pore water and sediment. Pore water is used for the analysis of nutrients and major ions, while sediments are used for the analysis of trace metals, rare earth elements (REEs), bacterial communities, and carbon and nitrogen isotopes. Two asphalt samples taken from dives #1345 and #1346 were also analyzed for carbon and nitrogen isotopes. We used these data to evaluate the asphalt seepage activity and to assess whether asphalt provides food for benthic animals.

## Results

### Description of sediment cores

Four sediment cores #1343H1 (20°42.6262′S, 38°38.1977′W), #1345H1(20°43.1341′S, 38°39.1038′W), #1346H1 and #1346H2 (20°43.9033′S, 38°39.7294′W) were taken from the northeast, middle and southwest areas of the asphalt seeps in the North São Paulo Plateau during dives #1343, #1345 and #1346, respectively (Figs 1b, 2d). The temperature, salinity, dissolved oxygen, visibility and water flow speed of the near-seabed water at these core sampling sites are ~2.6 °C, 34.9 PSU, 9.5 mg/L, 10–13 m, and 0.04–0.1 m/sec, respectively. Cores #1343H1, #1345H1 and #1346H2 were taken from outside of the asphalt areas without any obvious amount of asphalt mixed into them. #1346H1 is closely adjacent to the asphalt, and the surface sediment of 1346H1 is covered by solidified asphalt. Core #1340H1 (22°57.1972′S, 38°35.9570′W) taken from a non-asphalt seep area in the South São Paulo Plateau (Fig. 1c) was used only for comparing bacterial communities between asphalt seep sites and non-asphalt seep areas due to the limited number of samples.

### Nutrients and major ions in pore water and overlying seawater

**Figure 3:**
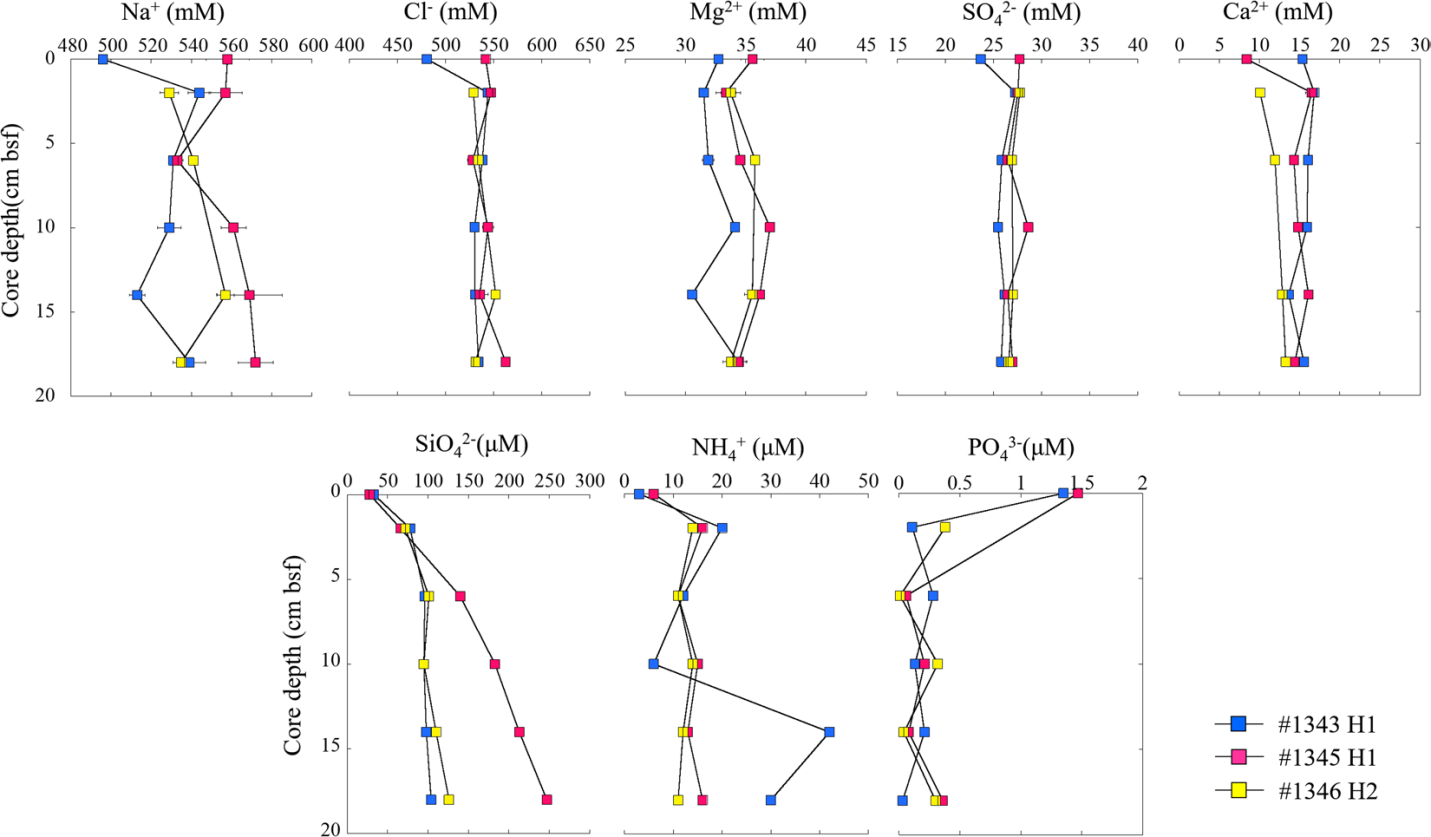
Vertical profiles of the major ion and nutrient concentrations in the pore water.

The data used in this study for the concentrations of nutrients and major ions in the pore water and overlying water are shown in Table 1 and Fig. 3. Silica concentrations in the pore water showed a progressive down-core increase. In cores #1343H1 and #1346H2, the silica concentration profiles were similar, ranging from 72 to 124 μM. Core #1345H1 showed a steep concentration gradient, with silica concentrations ranging from 66 to 247 μM. In all three cores, silica concentrations were higher than in the overlying seawater (28 to 33 μM). Ammonia and phosphate concentrations showed no significant relationship with the core depth. Ammonia in the overlying seawater was low (~6 μM) but increased in the pore water (11 to 42 μM). Phosphate in the overlying seawater was ~1.4 μM but decreased in the pore water (0.03 to 0.38 μM). Small deviations were observed for major ions in all three cores, and their concentrations were relatively constant at the different core depths. The average concentrations were Na^+^ (544 mM), Cl^−^ (539 mM), SO_4_^2−^ (27 mM), Mg^2+^ (34 mM), Ca^2+^ (14 mM), and K^+^ (9 mM). The ratios of Na^+^, Ca^2+^, Mg^2+^ and K^+^ to Cl^−^ were identical in the overlying seawater and the pore water of the three sites and did not vary significantly with core depth. However, an inconspicuous difference was observed at core #1345H1. The sodium concentration in core #1345H1 gradually increased with depth and was slightly higher than in cores #1343H1 and #1346H2.

**Table 1:** Nutrient and major ion concentrations in the pore water and overlying seawater, and carbon and nitrogen stable isotopes of sediments and asphalt.

Dive	Core depth	Na	Cl^−^	Mg	SO_4_^2−^	Ca	K	Si	NH_4_^+^	PO_4_^3−^	δ^13^C	δ^15^N
	cm bsf	mM	mM	mM	mM	mM	mM	μM	μM	μM	‰	‰
#1343	0	496	481	32.7	23.7	15.4	8.9	33	≤3	1.35	—	—
	0–4 cm	544	544	31.5	27.3	16.8	8.8	78	20	0.11	−20.3	7.2
	4–8 cm	531	538	31.9	25.9	16.0	9.1	96	12	0.28	−20.1	7.6
	8–12 cm	529	530	34.1	25.5	15.9	8.8	95	6	0.13	−20.3	7.8
	12–16 cm	513	531	30.6	26.2	13.7	7.9	98	42	0.21	−20.3	8.1
	16–20 cm	539	534	34.4	25.8	15.5	8.9	104	30	0.03	−20.7	7.9
#1345	0	558	542	35.6	27.7	8.4	8.7	28	6	1.47	—	—
	0–4 cm	557	547	33.4	27.6	16.5	11.7	66	16	—	−20.1	7.4
	4–8 cm	533	529	34.5	26.5	14.3	8.4	140	11	0.06	−21.3	7.2
	8–12 cm	561	544	37.0	28.6	14.8	9.3	183	15	0.21	−20.6	6.2
	12–16 cm	569	536	36.2	26.5	16.1	9.2	213	13	0.08	−20.3	7.0
	16–20 cm	572	562	34.4	27.0	14.4	9.1	247	16	0.36	−21.8	8.0
#1346	0	—	—	—	—	—	—	—	—	—	—	—
	0–4 cm	529	529	33.75	27.7	10.1	8.3	72	14	0.38	−19.9	7.2
	4–8 cm	541	534	35.78	26.9	11.9	8.5	101	11	0.01	−20.4	7.0
	8–12 cm	—	—	—	—	—	—	95	14	0.32	−20.9	7.8
	12–16 cm	557	552	35.55	27.0	12.9	10.4	110	12	0.04	−19.9	7.8
	16–20 cm	535	531	33.79	26.6	13.3	8.2	126	11	0.30	−21.9	8.0
#1345_asphalt	—	—	—	—	—	—	—	—	—	—	−25.8	0.5
	—	—	—	—	—	—	—	—	—	—	−26.3	0.4
	—	—	—	—	—	—	—	—	—	—	−26.9	0.3
#1346_asphalt	—	—	—	—	—	—	—	—	—	—	−27.3	1.3
	—	—	—	—	—	—	—	—	—	—	−27.8	1.2
	—	—	—	—	—	—	—	—	—	—	−28.4	1.1

No data.

### REE and trace metals distributions in sediments

**Figure 4:**
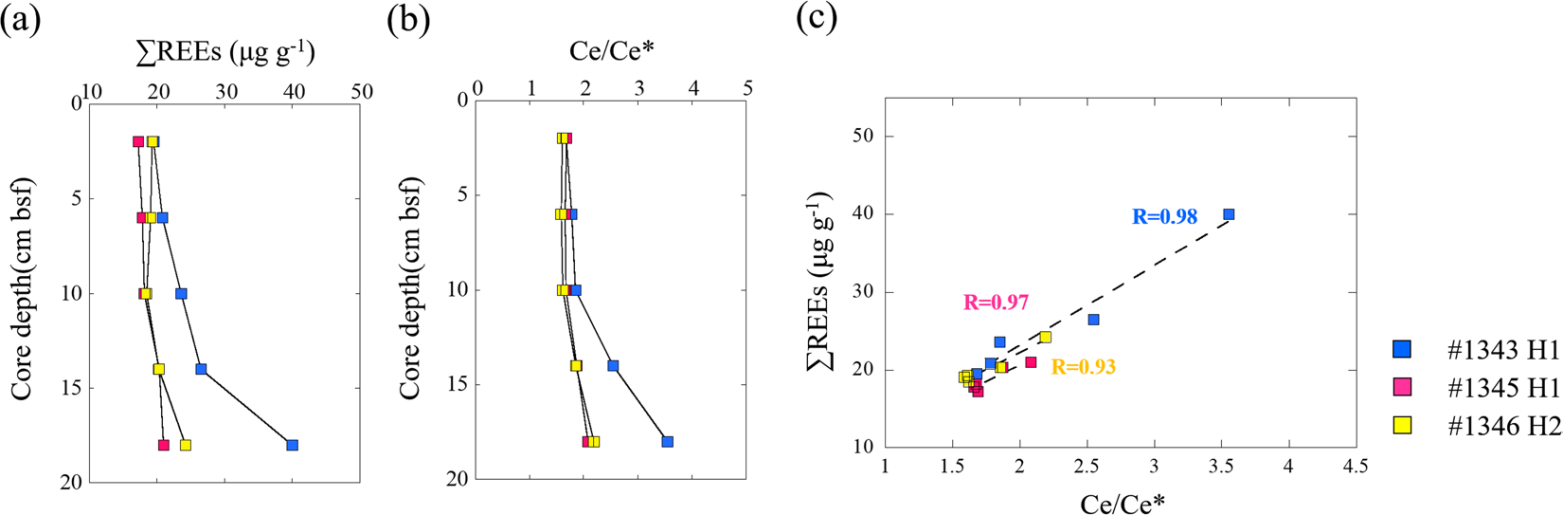
Signatures of the REE geochemistry in the Fe-Mn oxyhydroxide phases from the three asphalt seepage cores (#1343H1, #1345H1 and #1346H2). (**a**,**b**) Vertical profile of the ∑REE concentration and Ce anomaly. (**c**) Ce anomaly and ∑REE concentration correlations.

Table 2 records the distributions of rare earth elements in the sediment and the signatures of the REE geochemistry were shown in the Fig. 4. Three surface sediment samples from core #1343H1, #1345H1 and #1346H1 were chosen to represent the total rare earth elements (∑REE) in the bulk sediments. The ∑REE concentration ranged from 81.8 µg/g to 118.9 µg/g. The highest ∑REE concentration was detected in core #1343H1, while the lowest ∑REE concentration was detected in core #1346H1. Three sediment cores (#1343H1, #1345H1 and #1346H2) were analyzed to determine the ∑REE vertical profile in the Fe-Mn oxyhydroxide phase, which showed ∑REE concentrations ranging from 17.2 µg/g to 40.0 µg/g. In the Fe-Mn oxyhydroxide phase, core #1343H1 also showed the highest concentration of ∑REEs. Also in the Fe-Mn oxyhydroxide phase, the ∑REE concentration increased with depth among the three cores. The percentages of ∑REEs in the Fe-Mn oxide fraction were approximately 15% to 20% of the ∑REEs in the bulk sediments. The PAAS-normalized REE pattern in the bulk sediments showed a slight mid-REE enrichment. In the Fe-Mn oxide fraction, samples at the different core depths had similar relatively flat PAAS-normalized REE patterns, showing distinct positive Ce anomalies. The Ce anomaly became stronger with increasing depth.

**Table 2:** Trace metals and rare earth elements of sediments.

Dive		Core depth	Fe	Mn	Al	Zn	Cu	Y	La	Ce	Pr	Nd	Sm	Eu	Gd	Tb	Dy	Ho	Er	Tm	Yb	Lu	∑REEs	Ce_anomaly_
		cm bsf	(µg/g)																					
	B	0–4 cm	—	—	—	—	—	14.63	21.79	45.32	5.20	18.48	3.62	0.77	3.38	0.49	1.86	0.48	1.40	0.16	1.18	0.13	104.25	4.26
#1343	F	0–4 cm	3544	247	974	0.022	0.020	2.97	3.54	11.0	0.64	2.27	0.51	0.12	0.46	0.074	0.32	0.089	0.25	0.033	0.20	0.026	19.53	1.68
	F	4–8 cm	3558	245	939	0.022	0.023	3.13	3.62	12.0	0.67	2.34	0.51	0.13	0.49	0.079	0.35	0.095	0.27	0.036	0.22	0.027	20.83	1.78
	F	8–12 cm	3089	211	784	0.019	0.021	3.57	3.95	13.7	0.74	2.68	0.60	0.14	0.57	0.088	0.39	0.109	0.31	0.040	0.25	0.032	23.61	1.85
	F	12–16 cm	3564	230	1185	0.022	0.025	3.28	3.51	17.4	0.71	2.51	0.57	0.14	0.55	0.083	0.36	0.100	0.28	0.038	0.24	0.029	26.53	2.55
	F	16–20 cm	3900	232	1409	0.022	0.025	3.37	4.09	29.2	0.88	3.10	0.67	0.15	0.66	0.095	0.41	0.110	0.31	0.040	0.25	0.033	40.02	3.55
	B	0–4 cm	—	—	—	—	—	11.23	17.32	36.96	4.16	14.78	2.90	0.62	2.69	0.38	1.46	0.37	1.08	0.12	0.90	0.10	83.84	4.36
#1345	F	0–4 cm	2667	168	719	0.017	0.015	2.60	3.09	9.7	0.57	2.02	0.45	0.11	0.43	0.070	0.30	0.082	0.22	0.029	0.18	0.023	17.24	1.69
	F	4–8 cm	2930	196	729	0.018	0.017	2.77	3.21	10.0	0.60	2.12	0.47	0.12	0.45	0.072	0.31	0.086	0.24	0.031	0.19	0.025	17.86	1.66
	F	8–12 cm	2905	197	702	0.017	0.019	2.84	3.25	10.2	0.60	2.15	0.49	0.12	0.45	0.070	0.32	0.087	0.24	0.031	0.19	0.024	18.18	1.68
	F	12–16 cm	3404	214	893	0.019	0.020	3.08	3.19	11.9	0.67	2.42	0.55	0.13	0.51	0.083	0.34	0.093	0.26	0.035	0.20	0.027	20.36	1.87
	F	16–20 cm	3798	1285	880	0.047	0.022	2.94	3.07	12.8	0.65	2.37	0.53	0.13	0.49	0.077	0.33	0.090	0.25	0.033	0.20	0.025	21.00	2.08
	B	0–4 cm	—	—	—	—	—	9.62	14.35	32.38	3.53	12.71	2.54	0.54	2.36	0.34	1.28	0.32	0.92	0.10	0.75	0.08	72.19	4.55
#1346	F	0–4 cm	3203	246	860	0.047	0.019	3.19	3.52	10.6	0.66	2.37	0.51	0.13	0.49	0.081	0.33	0.092	0.26	0.034	0.20	0.026	19.31	1.61
	F	4–8 cm	4081	285	1121	0.025	0.024	3.14	3.59	10.5	0.65	2.26	0.49	0.13	0.47	0.075	0.33	0.091	0.26	0.033	0.20	0.027	19.08	1.59
	F	8–12 cm	3263	241	874	0.021	0.024	3.10	3.44	10.3	0.62	2.17	0.48	0.11	0.45	0.073	0.31	0.090	0.25	0.034	0.20	0.026	18.51	1.62
	F	12–16 cm	3389	248	1004	0.021	0.026	3.15	3.43	11.9	0.64	2.24	0.50	0.12	0.47	0.076	0.33	0.091	0.25	0.033	0.21	0.026	20.32	1.86
	F	16–20 cm	3042	216	967	0.019	0.022	3.25	3.68	15.2	0.70	2.44	0.52	0.13	0.50	0.079	0.33	0.094	0.26	0.034	0.21	0.027	24.26	2.19

No data; B: bulk sediment; F: Fe-Mn oxyhydroxide phases.

The concentrations of trace metals in the Fe-Mn oxyhydroxide phase were 2600 to 4100 µg/g for Fe, 160 to 290 µg/g for Mn, 700 to 1400 µg/g for Al, 0.01 to 0.05 µg/g for Zn, and approximately 0.2 µg/g for Cu. These values showed no significant changes with depth, except for Fe and Mn in core #1345H1, the concentrations of which slightly increased with depth. Overall, the trace metal concentrations were about the same in the three cores, although that of core #1345H1 were slightly lower than those of #1343H1 and #1346H2.

### Bacterial communities in sediments

**Figure 5:**
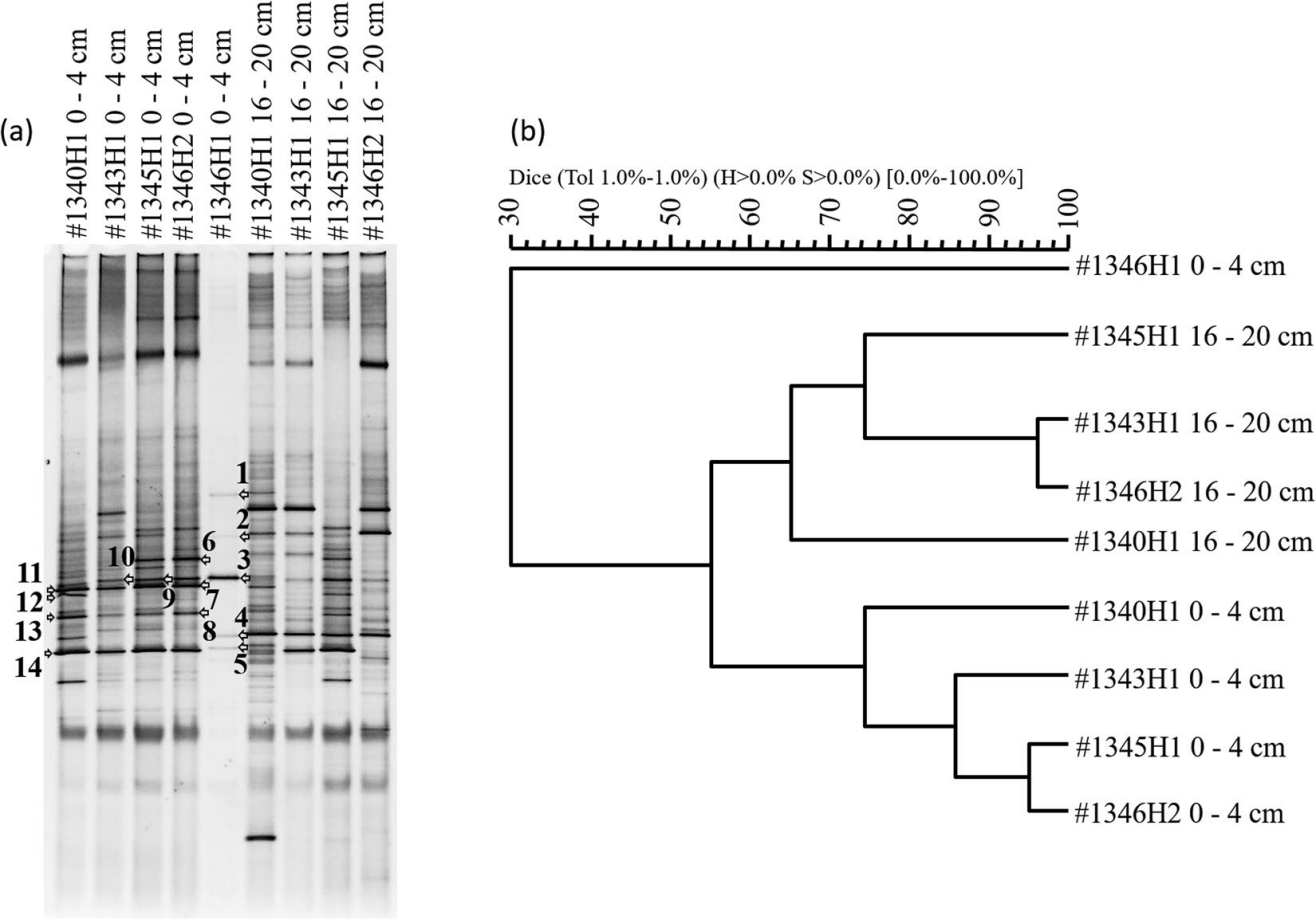
(**a**) Denaturing gradient gel electrophoresis (DGGE) profiles of bacterial communities at different sites. Arabic numerals indicate successfully sequenced bands for bacteria. (**b**) Dendrogram of bacterial communities at different sites. An unweighted pair group method with arithmetic average (UPGMA) dendrogram was constructed from Fig. 5(a) to show the similarity of the lanes of the DGGE profile.

The bacterial communities in sediment cores #1340H1, #1343H1, #1345H1, #1346H1 and #1346H2 were determined by the PCR-DGGE method. Core #1340H1 was taken from a non-asphalt seep area in the South São Paulo Plateau, and the surface sediment of core #1346H1 is mixed with solidified asphalt. These samples represent bacterial communities in non-asphalt sediment and asphalt, respectively. Figure 5 shows the denaturing gradient gel electrophoresis (DGGE) profiles of bacterial communities in the different samples. Arabic numbers are used to mark the DGGE gels of successfully sequenced bands. The GenBank sequences of the 16S rRNA genes for bacteria were aligned with these sequences, and the results are listed in Table 3. The results show that the bacterial domains exhibited distinct communities at the different core depths. The diversity of bacteria in the surface sediment of #1346H1 was much lower than that in the other sediment cores. Furthermore, in the surface sediment of #1346H1, the predominant bacterial groups were Proteobacteria, while in samples #1340H1 and #1343H1, the predominant bacterial groups were Firmicutes. The predominant bacterial groups detected in #1345H1 and #1346H2 were Proteobacteria and Firmicutes. The main bacteria in the surface sediment of #1346H1, marked by Arabic numeral 3 in the DGGE profiles (Fig. 5a), was 100% similar to the organism of *Erythrobacter citreus* strain VSW309, with GenBank accession No. KC534372. This species of bacteria was first detected in the hydrothermal vent of Espalamaca^17^. Another bacterial species, marked by Arabic numeral 5 in DGGE profiles (Fig. 5a), was 98% similar to *Thalassospira xianhensis* strain MT02, with GenBank accession No. KX180910. *Thalassospira xianhensis* sp. nov is a hydrocarbon-degrading marine bacterium^18^. Furthermore, symbiotic bacteria first found in a sponge in the Persian Gulf were also detected in the sediment of #1345H1 and #1346H2 (GenBank accession No. KY283118). These three species of bacteria indicate that bacteria might play an important role in the benthic ecosystem. The similarity of the lanes of the DGGE profiles is shown by a dendrogram of the bacterial communities at different sites (Fig. 5b). Comparing the bacterial communities in cores #1343H1, #1345H1, and #1346H2 with those in #1340H1, the similarity of the bacterial patterns was 74% in the surface sediment (0–4 cm) and 65% in the bottom sediment (16–20 cm). The bacterial patterns in the surface sediments (0–4 cm) of #1345H1 and #1346H2 were close, showing 95% similarity. In addition, the bacterial diversity and biomass of these samples was higher than those at #1343H1. Conversely, in the bottom layer (16–20 cm), the bacterial pattern similarity of #1343H1 and #1346H2 was 96%, showing a great difference from #1345H1. The main bacterial species of #1345H1 seemed to be more complex than those of #1343H1 and #1346H2.

**Table 3:** Bacterial sequences identified in #1340H1, #1343H1, #1345 H1, #1346H1 and #1346 H2 and their closest matches to the 16 S rRNA gene sequences of the bacteria database in GenBank.

Band			Closest match	
No.	Phylum	Similarity	Organism	Accession No.
1	Proteobacteria	99%	*Pseudomonas* sp. 01WB02.2–34	FM161390
2	Proteobacteria	75%	*Pseudomonas thivervalensis* HMGU204	HF952551
3	Proteobacteria	100%	*Erythrobacter citreus* strain VSW309	KC534372
4	Proteobacteria	98%	*Sulfitobacter* sp. TJD639	EF364127
5	Proteobacteria	98%	*Thalassospira xianhensis* strain MT02	KX180910
6	Proteobacteria	100%	*Psychrobacter* sp. strain m.zarei	KY283118
7	Firmicutes	99%	*Bacillus* sp. HSS-410	FN674519
8	Firmicutes	100%	*Bacillus* sp. 3–10 (2012)	KC527675
9	Firmicutes	97%	*Fictibacillus phosphorivorans* strain GYXG-9	MF101197
10	Firmicutes	97%	*Bacillus* sp. 2049	JX566560
11	Firmicutes	97%	*Fictibacillus phosphorivorans* strain GYXG-9	MF101197
12	Firmicutes	97%	*Bacillus* sp. CAU 1339	KX768749
13	Firmicutes	98%	*Bacillus* sp. CAU 1339	KX768749
14	Firmicutes	95%	*Bacillus* sp. CAU 1339	KX768749

### Carbon and nitrogen stable isotopes

The carbon and nitrogen stable isotope results from the sediment organic matters (SOM) and asphalt are listed in Table 1. The δ^13^C and δ^15^N values of SOM ranged from −22.5‰ to −19.9‰ and 6.2‰ to 8.0‰, respectively. The δ^13^C and δ^15^N values of asphalt ranged from −26.9‰ to −25.8‰ and 0.3‰ to 0.5‰, respectively, in dive #1345 and from −28.4‰ to −27.3‰ and 1.1‰ to 1.3‰, respectively, in dive #1346.

## Discussion

This is the first discovery of asphalt seeps located in the Brazilian margin and the Southeastern Atlantic. The escarpment, a 200-m relief on the seafloor, suggests an active fault system related to salt diapirism^16^. Asphalt seeps discovered along the escarpment indicate that the salt diapirism is connected to deep oil reservoirs or to the source rock. The fault may provide a pathway for hydrocarbons to migrate around the margins of the salt diapirism and seep out onto the seafloor. The petroleum systems described here are similar to the asphalt sites of the Campeche Knolls in the southern Gulf of Mexico^8^ and the Angolan margin^11^. They are probably controlled by the movement of salt structures^11,16,19^. The asphalt in this study is most comparable to that found in the Angolan margin. These two asphalt seep sites, located at opposite Atlantic margins, appear less active than those in the Gulf of Mexico and the Santa Barbara Basin. Non-chemosynthetic faunae are positively correlated with the distribution of asphalt. Similar habitats, such as those nurturing porifera, chordata, arthropoda, and cnidaria, were observed in both areas^11,14^. Furthermore, the oceanographic conditions and depositional environments at these two margins are nearly identical, and their geologies are well matched in age, lithology and sub-bottom depths^13^. A reconstruction of the South Atlantic at the end of the Aptian era shows that the asphalt sites at the North São Paulo Plateau were adjacent to the asphalt sites at the Angolan margin^15^. However, the data from the asphalt we collected from the North São Paulo Plateau are limited. Nevertheless, the high C35 homohopane index of this asphalt indicates that its origin is a carbonate source rock^16^. The δ^13^C and δ^15^N values of the asphalt suggest that it is mainly terrigenous organic matter^14^. Currently, no related data have been reported about the asphalt in the Angolan margin. Further research on these two asphalt sites of the São Paulo Plateau and the Angolan margin may provide useful information to further understanding of the evolutionary history of the northern South Atlantic.

A distinct asphalt feature of the North São Paulo Plateau is that no chemosynthetic communities, bacterial mats, gas seepage (bubbles) or pockmarks were observed during the surveys. The asphalt is solidified, and no fresh oil was observed, implying that the asphalt seeps in this study are plausibly inactive. To further confirm the activity of the asphalt seeps, we measured the nutrients and major ions in the pore water and the REEs and bacterial communities in the sediments. In marine environments, the pore water chemistry of sediments is a reflection of various biogeochemical processes^20^. Different from sediment chemistry, which in most cases represents past events, the chemistry of pore water can provide more real-time and dynamic information. In the active seep sites, fluid seeps out and directly influences the chemical distribution in the pore water^20,21^. In this study, the most distinctive characteristic of the pore water was that dissolved silica showed steep concentration gradients in sample #1345H1—the concentration at a core depth of 16–20 cm below seafloor (bsf) was as high as 247 µM, which was more than twice as high as that in #1343H1 and #1346H2. Two potential mechanisms could contribute to the increase of dissolved silica in pore water. One is the diagenesis of the smectite-to-illite transformation, producing an important byproduct as dissolved silica^22^. The other mechanism is the high concentration of salt in brine effectively leaching silica from inorganic matter^23^. Both of these mechanisms are related to the thermal gradient, but we cannot determine which is the main cause of the elevated silica in the pore water. It seems that the process of silica enrichment happens below core depth. The smectite-to-illite transformation process could have led to the decrease in potassium and increase in magnesium in the pore water; however, no significant correlation was observed among silica, potassium and magnesium. Moreover, the total cation concentration (sodium, potassium, calcium and magnesium) at #1345H1 (624 mM) was only approximately 5–6% higher than that at #1343H1 (590 mM) and #1346H2 (596 mM), making the cation concentration an unlikely contributor to the leaching of silica from inorganic matter. However, the elevated sodium concentration at the greater core depth in #1345H1 indicates that the fluid seeps upward with enriched silica. A similar silica distribution trend was also observed at two active seafloor brine sites in the Gulf of Mexico^24^. At these two reference sites, the same level of silica concentration was observed in the bottom water and increased to over 200 µM at the depth of 60–100 cm bsf, and the temperature of the brine was ≧10 °C higher than the ambient seawater. Dissolved silica showed a steeper concentration gradient in this study compared with those found at the two active brine sites in the Gulf of Mexico. One potential explanation for the high concentration in #1345H1 is that there is an active oil field brine below the surface sediment.

Sulfate in pore water is a good indicator of methane or oil seepage. In general, the consumption of methane and the biodegradation of oil are closely related to the distribution of sulfate^25^ and promote early diagenesis in pore water. One of the most conspicuous processes in this regard is the authigenic formation of carbonate minerals. The cold seep at Hydrate Ridge is a typical representative, showing distinct sulfate and calcium consumption^21^. At the three sampling sites in this study (#1343H1, #1345H1 and #1346H2), the variations of sulfate and calcium in the pore water were minimal, and they showed no significant correlation between each other. In deep oil reservoirs, the density of methane and oil is lower than that of brine water, and as a result, these materials will seep out first. Thus, it is possible that methane and asphalt in deep oil reservoirs was released during the active period of the asphalt seeps, indicating active oil field brine below the surface sediment. The current methane seepage and asphalt biodegradation activities are too weak to alter the distribution of sulfate and calcium in the pore water. Another possibility is that samples were taken from sediment of the surface at 20 cm, a depth which is easily influenced by bottom seawater, leading to the relative constant sulfate and calcium concentrations in the pore water.

Further discussion is focused on the REEs data from the sediments. Previous studies have shown that crude oil is a potential additional source of REEs at oil seeps^26,27^ because REEs are preferentially bound to metalloporphyrines in natural oils^28^ when the crude oils are generated^26^. Our results show that the bulk surface sediment of core #1346H1, which included a high percentage of solidified asphalt, had a relatively low concentration of REEs compared to those of core samples #1343H1 and #1345H1. One explanation is that the metalloporphyrines of the asphalt are very low, which led to low concentration of REEs in the asphalt during the oil fractionation process. Additionally, it is also possible that REEs had been released from the asphalt into the surrounding environment during the asphalt biodegradation process. PAAS-normalized REE patterns of the Fe-Mn oxyhydroxide phase showed middle-REE enrichment with a strongly positive cerium (Ce) anomaly. This is typical of sedimentary Fe-Mn oxyhydroxide phases^29^ and similar to REE patterns of the hydrogenous ferromanganese crust on the Sao Paulo Ridge^30^. Ce is a redox-sensitive element. It has trivalent and quadrivalent valences. Under oxidation conditions, it can be easily oxidized in liquid and absorbed into particles, resulting in the positive Ce anomaly in the sediment. This suggests that REEs in the Fe-Mn oxyhydroxide phase had a similar source to that of the REEs of the hydrogenous ferromanganese crust on the Sao Paulo Ridge, which are mainly from seawater^31^. A relatively lower ∑REE concentration and weakly positive Ce anomaly were observed in cores #1345H1 and #1346H2, compared to those of core #1343H1 (Fig. 4a,b). The Fe-Mn oxyhydroxide phase is sensitive to the redox condition of the sediment. Under oxidizing conditions, dissolved REEs are easily scavenged from liquid due to co-precipitation of the Fe-Mn oxyhydroxide phase, while under reducing conditions, the Fe-Mn oxyhydroxide phase of dissolved sediment and REEs and trace metals are released into the pore water^32,33^. Our results imply that when the asphalt seeps were active, reducing fluid from the oil reservoir lead to REEs being released from the Fe-Mn oxyhydroxide phases of the sediments into the pore water.

In addition, the bacterial communities in sediments also seem to be influenced by the asphalt seeps. Bacterial communities in sediments are mainly determined by the sedimentary environment and chemical matter. Bacteria in the asphalt that could be detected by DGGE belonged to Proteobacteria, while in the non-asphalt seep site (#1340H1), bacteria that could be detected by DGGE belonged to Firmicutes. In the surface sediment (0–4 cm) of asphalt seep sites, the bacterial patterns of #1345H1 and #1346H2 were similar, and their diversity and biomass of bacteria were higher than those of #1343H1. Both Proteobacteria and Firmicutes were detected in #1345H1 and #1346H2, while only Firmicutes were detected in the surface sediment of #1343H1, showing a type of non-asphalt seep bacterial community. This indicates that bacterial communities in surface sediments #1345H1 and #1346H2 were possibly affected by asphalt, while the effect of asphalt on the bacterial communities in the surface of #1343H1 was inconspicuous. Interestingly, in the deeper layer (16–20 cm), the bacterial communities were greatly different. Site #1345H1 is located between #1343H1 and #1346H2, but the bacterial patterns of #1343H1 and #1346H2 were highly similar, and the main bacterial species were significantly different from those in #1345H1. One possible explanation is that active oil field brine flows upward, transporting chemicals to core #1345H1 and changing the sedimentary environment and chemical composition in the sediment, resulting in different bacterial communities. However, it is unlikely that only two phyla of bacteria exist in the environmental samples. Because DGGE can detect only the dominant bacteria, in the future, it is necessary to analyze the communities in more detail using a next-generation sequencer.

**Figure 6:**
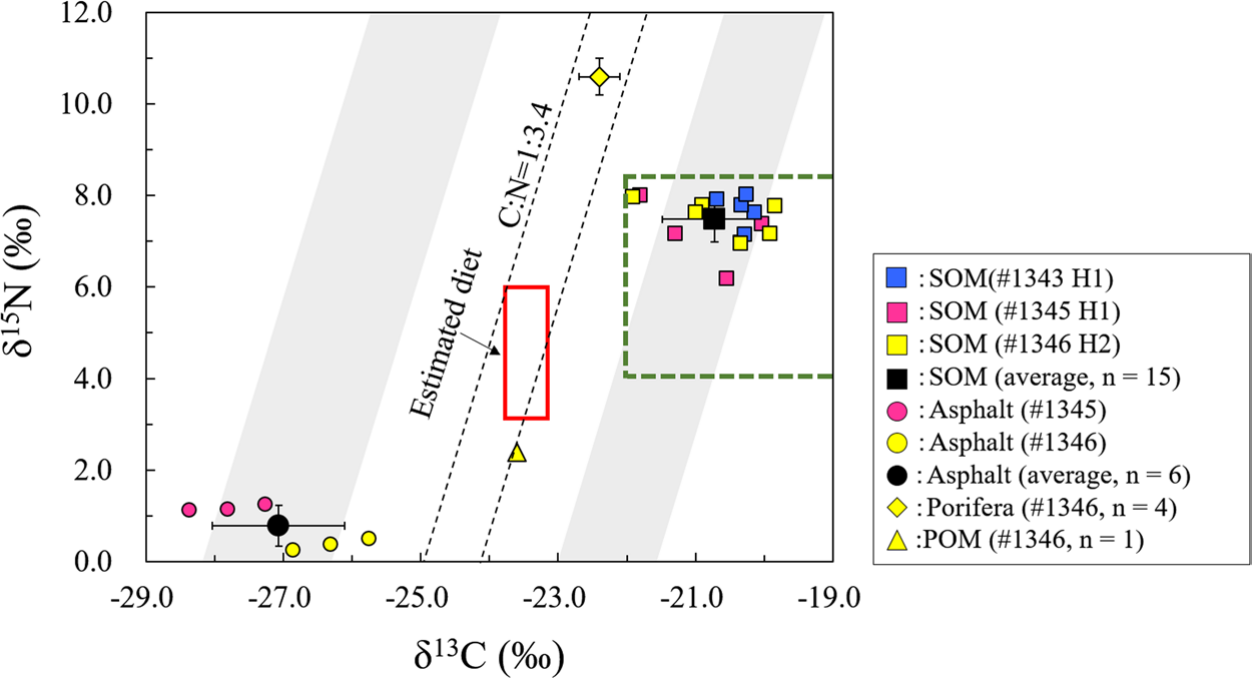
Distribution of carbon and nitrogen stable isotope ratios among porifera, asphalt, organic matters in sediments (SOM) and particulate organic matter (POM). The green dashed square presents the range of δ^13^C and δ^15^N values of marine-origin sources in the South Atlantic Ocean^34–36^, the black dashed box represents the theoretical isotopic shift in the food chain of porifera^37,38^, the gray boxes represent the theoretical isotopic shift from SOM and asphalt to their consumes, and the red square represents the range of δ^13^C and δ^15^N values of porifera’s estimated feed (porifera and POM data come from Fujikura *et al*.^14^).

Another topic of this study is whether an asphalt seep could provide a carbon source for benthic animals. Non-methane seepage occurs in our newly formed asphalt seeps, and pure asphalt seeps compel us to study the influence of heavy hydrocarbon seepage on benthic ecosystems. According to field observations, many benthic animals were observed in the asphalt seep sites (Fig. 2e,f). Sponges, polichaetes, tunicates, shrimps, sea cucumbers, anemones, brittle star, starfish, squat crab, hydrozoans, octopuses and fishes were observed. In particular, sponges were observed in the presence of asphalt (for further detailed information, see Fujikura *et al*.,^14^). Like other asphalt sites in the Gulf of Mexico and the Angolan margin, the asphalt at these sites appears to provide a hard substratum for sessile fauna. However, interesting findings were obtained through analysis of the stable carbon and nitrogen isotope data. Stable carbon and nitrogen isotopes are good tracers for understanding the food sources of heterotrophs and trophic pathways within food webs. Figure 6 presents the δ^13^C and δ^15^N values of porifera, asphalt, SOM and particulate organic matter (POM). The orange square shows the range of δ^13^C and δ^15^N values of the marine-origin source in the South Atlantic Ocean^34–36^, and the δ^13^C and δ^15^N values of organic matter in the SOM were inside this range. This suggests that the SOM mainly originated from organic matter in the seawater^14^. According to these results, the δ^13^C and δ^15^N values of porifera are closer to those of the SOM. It has been reported that stable carbon and nitrogen isotopes display an isotopic shift from diet to consumer during the food-assimilation and waste-excretion processes. Typically, the mean isotopic shifts for δ^13^C and δ^15^N are approximately 0.5–1.0‰ and 2–5‰, respectively^37,38^. If we assume that the food source of porifera is only SOM, according to the isotopic shift in the food chain^37,38^ and the δ^13^C value of SOM (−20.8 ± 0.8‰), the theoretical δ^13^C value of porifera should be around −21.1 to −19.0‰, which is much higher than our detected value of −22.4‰. Sponges that prey on marine organic matter show a δ^13^C value ranging from −17.2‰ to −20.7‰^39^. This implies that the carbon source of porifera also came from other organic matter with low values of δ^13^C. As discussed above, the SOM mainly originated from seawater, so the δ^13^C and δ^15^N values of the POM and SOM should be similar. However, Fig. 6 shows that the δ^13^C and δ^15^N values of the POM were between those of the asphalt and SOM. This implies that the POM is influenced by suspended asphalt particles. Marine sponges are filter-feeding animals^40^ and feed on organic matter by drawing water in through their pores. Supposing that both SOM and asphalt provide a carbon source for porifera, a two end-member mixing model^41^ was used for the fractional contributions of the two carbon sources. Animals usually have higher δ^13^C values than those of their diets, so we subtracted 1‰ from the δ^13^C value of −22.4‰ for porifera. Consequently, the δ^13^C value of the porifera diet was estimated as −23.4‰. The fractional contribution of the carbon sources from asphalt was calculated according to the following equation:

1 $$ \% \,{\rm{Asphalt}}\,{\rm{C}}=[1-({{\rm{\delta }}}^{13}{{\rm{C}}}_{porifera}-{{\rm{\delta }}}^{13}{{\rm{C}}}_{asphalt})/({{\rm{\delta }}}^{13}{{\rm{C}}}_{som}-{{\rm{\delta }}}^{13}{{\rm{C}}}_{{ashphalt}})]\times 100$$

where % Asphalt C is the percentage of carbon source from asphalt, δ^13^C_porifera_ is the δ^13^ value of porifera with a fractionation correction of 1‰, δ^13^C_asphalt_ is the δ^13^ value of asphalt, and δ^13^C_SOM_ is the δ^13^ value of SOM. The values of δ^13^C_porifera,_ δ^13^C_Asphalt,_ and δ^13^C_SOM_ were −23.4 ± 0.3‰ (n = 4), −27.1 ± 1.0‰ (n = 6), and −20.8 ± 0.8‰ (n = 15), respectively. This model estimates that the asphalt provided 29–56% of the carbon source for porifera. Based on this estimated result, we can also calculate that the δ^15^N value of the porifera diet ranges from 3.3 to 6.0‰. This shows an isotopic shift of one trophic level within the uncertainties, compared with the δ^15^N value of porifera (10.6 ± 0.4‰) (Fig. 6). As in this study, heavy hydrocarbon providing a carbon source for ecosystems has also been observed at several petroleum seepages^42,43^. For example, the δ^13^C value of plankton in a water column significantly decreased during the Deepwater Horizon oil spill compared with that before the accident^43^. Although the evidence for oil providing a carbon source for ecosystems is inadequate and little is known about how such oil carbon currently enters the food web, the important impact of asphalt on the ecosystem cannot be neglected.

In conclusion, it should be noted that the asphalt seeps were located on the sea floor at a depth of 2700 meters, so they are very difficult to reach. This was the first deep-sea survey conducted there using a manned submersible, and only limited samples were taken from the asphalt seep sites due to the storage constraints of SHINKAI 6500. Perhaps these samples cannot fully reflect all characteristics of the asphalt seeps. Notwithstanding this limitation, this study acquired the first multiple biogeochemical data from asphalt seeps in the Brazil margin, which could be a valuable addition to research on asphalt seeps in the world. Additionally, this study may provide useful information to further understanding of the evolutionary history of the northern South Atlantic and clarify the related impact of heavy hydrocarbon seepage on the marine ecosystem.

## Methods

### Sample collection and pretreatment

All samples used in this study, including sediment cores and asphalt, were taken using the human-driven vehicle SHINKAI 6500 during the Iatá-piúna cruise in May 2013. Sediment cores were continuously sliced at 4-cm intervals while on board. Immediately thereafter, pore water was squeezed out by pressure filtration through a 0.45-µm membrane filter at 4 °C. Overlying seawater samples were also filtered through a 0.45-µm membrane filter. All water samples were stored in a refrigerator at 4 °C for analysis. Sediment and asphalt were stored in a freezer at −20 °C. The methods used in this study are described as follows.

### Nutrient measurement

Pore water and overlying seawater samples were analyzed for silica and ammonia on board by colorimetric techniques immediately after recovering the samples on deck and squeezing the pore water from the core sediments. The molybdenum blue method was used for silica, and the indophenol method was used for ammonia, as described in Gieskes *et al*.^44^. Phosphates were determined by a molybdenum blue method in a laboratory on land using a UV–VIS spectrophotometer (SHIMADZU UVmini-1240).

### Major ions measurement

Major ions (Na^+^, K^+^, Mg^2+^, Ca^2+^, Cl^−^ and SO_4_^2−^) in the pore water and overlying seawater samples were measured in the laboratory on land. All samples were diluted approximately 500 times before measurement and then analyzed using ion chromatography (Metrohm 761 Compact IC) with RSD <3%.

### Rare earth element and trace metal analysis

Rare earth element and trace metal concentrations in the Fe-Mn oxide phase of sediment were analyzed by a chemical leaching method^45^. Sediment samples of 25 mg were processed in 50-ml acid-cleaned centrifuge tubes. The first step was removal of exchangeable fractions by adding 8 ml of 1 M MgCl_2_ with continuous agitation for 1 hour at room temperature. After centrifugation, samples were rinsed with deionized water, and the supernatant was decanted. Then, residue from the first step was added to 8 ml of 1 M NaOAc and adjusted to pH 5.0 with acetic acid and continuous agitation for 5 hours at room temperature for removal of the carbonate fraction. Again, the samples were centrifuged and rinsed with deionized water, and the supernatants were decanted. The residue was used for extraction of the Fe-Mn oxyhydroxide phase. Then, 20 ml of 0.04 M hydroxylamine hydrochloride in 25% (v/v) acetic acid was added, and the samples were heated in a water bath at 96 ± 3 °C for 6 hours. The supernatant was transferred to 20-ml Teflon bottles. Bulk sediments were processed by a total digestion method using a mixture of the acids HF, HNO_3_, and HClO_4_. Finally, all samples were dried on a hot plate at 135 °C, dissolved in 2% HNO_3_, and then analyzed by ICP-MS^46^ (HP 4500, RSD < 3%).

In this paper, the Ce anomaly was calculated according to the following equation:

2 $${{\rm{Ce}}}_{\mathrm{anomaly}}=C{e}_{N}/\sqrt{L{a}_{N}\times P{r}_{N}}$$

where N refers to normalization of the concentration against that of the Post Archean Australian Shale (PAAS)^47^.

### Determination of bacterial communities in sediment

Determination of the bacterial communities was performed using the PCR-DGGE method. The detailed experimental procedures and DEEE pattern analysis were the same as those described by Tiodjio *et al*.^48^. The sequences obtained from this study were deposited in the GenBank/EMBL/DDBJ databases under the accession numbers LC312654 to LC312667 for bacteria.

### Carbon and nitrogen stable isotopes

Stable isotopes (δ^13^C and δ^15^N) were determined by an automatic elemental analyzer (EuroVector EA3000) coupled with an isotope ratio mass spectrometer (IsoPrime, GV Instruments, Manchester, UK) (sediment and asphalt samples taken from dives #1345 and #1346) and an automatic elemental analyzer (Flash EA 1112, Thermo Fisher Scientific, UK) coupled with an isotope ratio mass spectrometer (Delta V Advantage, Thermo Fisher Scientific, UK) (sediment samples taken from dive #1343), respectively. The analytical precision was greater than ± 0.2%. Detailed procedures were reported by Fujikura *et al*.^14^.

## Data Availability

All data generated or analyzed during this study are included in this published article (and its Supplementary Information files).
